# Comparative assessment of diverse strategies for malaria vector population control based on measured rates at which mosquitoes utilize targeted resource subsets

**DOI:** 10.1186/1475-2875-13-338

**Published:** 2014-08-28

**Authors:** Gerry F Killeen, Samson S Kiware, Aklilu Seyoum, John E Gimnig, George F Corliss, Jennifer Stevenson, Christopher J Drakeley, Nakul Chitnis

**Affiliations:** Environmental Health and Ecological Sciences Thematic Group, Ifakara Health Institute, Ifakara, Kilombero, Morogoro, United Republic of Tanzania; Liverpool School of Tropical Medicine, Vector Biology Department, Pembroke Place, Liverpool, L3 5QA UK; Centers for Disease Control and Prevention, Division of Parasitic Diseases and Malaria, Atlanta, Georgia 30333 USA; Department of Electrical and Computer Engineering, Marquette University, Milwaukee, WI 53201-1881 USA; Department of Immunology and Infection, Faculty of Infectious and Tropical Diseases, London School of Hygiene and Tropical Medicine, Keppel Street, London, WC1E 7HT UK; Johns Hopkins Bloomberg School of Public Health, Johns Hopkins Malaria Research Institute, Baltimore, MD 21205 USA; Department of Epidemiology and Public Health, Swiss Tropical and Public Health Institute, Basel, Switzerland; University of Basel, Basel, Switzerland; Fogarty International Center, National Institutes of Health, Bethesda, MD 20892 USA

**Keywords:** *Plasmodium*, *Anopheles*, Vector control, Mosquito, Malaria, Target product profile

## Abstract

**Background:**

Eliminating malaria requires vector control interventions that dramatically reduce adult mosquito population densities and survival rates. Indoor applications of insecticidal nets and sprays are effective against an important minority of mosquito species that rely heavily upon human blood and habitations for survival. However, complementary approaches are needed to tackle a broader diversity of less human-specialized vectors by killing them at other resource targets.

**Methods:**

Impacts of strategies that target insecticides to humans or animals can be rationalized in terms of *biological coverage* of blood resources, quantified as proportional coverage of *all blood resources* mosquito vectors utilize. Here, this concept is adapted to enable impact prediction for diverse vector control strategies based on measurements of *utilization rate*s for *any definable, targetable resource subset*, even if that overall resource is not quantifiable.

**Results:**

The usefulness of this approach is illustrated by deriving utilization rate estimates for various blood, resting site, and sugar resource subsets from existing entomological survey data. Reported impacts of insecticidal nets upon human-feeding vectors, and insecticide-treated livestock upon animal-feeding vectors, are approximately consistent with model predictions based on measured utilization rates for those human and animal blood resource subsets. Utilization rates for artificial sugar baits compare well with blood resources, and are consistent with observed impact when insecticide is added. While existing data was used to indirectly measure utilization rates for a variety of resting site subsets, by comparison with measured rates of blood resource utilization in the same settings, current techniques for capturing resting mosquitoes underestimate this quantity, and reliance upon complex models with numerous input parameters may limit the applicability of this approach.

**Conclusions:**

While blood and sugar consumption can be readily quantified using existing methods for detecting natural markers or artificial tracers, improved techniques for labelling mosquitoes, or other arthropod pathogen vectors, will be required to assess vector control measures which target them when they utilize non-nutritional resources such as resting, oviposition, and mating sites.

**Electronic supplementary material:**

The online version of this article (doi:10.1186/1475-2875-13-338) contains supplementary material, which is available to authorized users.

## Background

While antiparasitic drugs and vaccines will be essential to the final stages of malaria elimination, their effectiveness as transmission control interventions will rely heavily upon unprecedented levels of vector control in highly endemic settings [1–4]. It will not be possible to eliminate malaria transmission from most of the tropics without developing scalable vector control intervention options which complement long-lasting insecticidal nets (LLINs) and indoor residual spraying (IRS) by targeting adult mosquitoes when they use resources other than human blood indoors, and indoor resting sites [5–9]. Mosquitoes usually also exploit non-human blood and human blood sources outdoors, as well as sugar, outdoor resting sites, oviposition sites, and mating sites, so all of these other biological and environmental resources represent alternative targets for vector control interventions [5].

Faced with such an array of resource target options, the challenge is to define exactly which of these intervention targets are optimal in each of the diverse vectorial systems that exist [10], and to classify these settings into limited numbers of distinct categories where specific intervention combinations maximize impact. Recent analyses indicate that the impact of vector control measures targeting the blood hosts upon which mosquitoes depend can be rationalized in terms of measurements of the *biological coverage* of *all* available blood sources that is achieved, rather than merely high *demographic* coverage of a *targeted subset*, such as humans while asleep indoors [9, 11]. Blood resources are perhaps the best understood, and most readily quantified, of all the resources used by mosquitoes that could potentially be targeted with vector control measures [5]. However, many established or emerging vector control strategies only target a specific subset of the resource that can be readily identified and treated in the field. Examples include targeting insecticides to indoor resting sites only with IRS [12], and artificially introduced resting sites [13–17] or sugar baits [18–20]. Furthermore, the other available forms of these resources that cannot be targeted with insecticides but compete with these subsets for the attentions of mosquitoes, such as naturally occurring sugar sources [21] or outdoor resting sites [22, 23], are often impossible to identify or quantify [5]. It is therefore not possible to estimate biological coverage as a fraction of all available forms of that resource. Here, the concept of biological coverage is extended beyond blood resources, and adapted to enable impact prediction for more diverse vector control strategies, based on direct measurements of *coverage* and *utilization rates* for *definable, targetable subsets* of less readily quantified resources that are equally important to mosquito survival, and therefore equally valid as potential targets for vector control interventions.

## Methods

All symbols used are listed and defined in Table 1.

**Table 1:** **Parameter symbols and definitions**

Symbol	Definition
*Previous formulations to predict impact based on of biological coverage of all blood resources*	
*A*	Availability of all blood hosts for attack, expressed as the rate at which they are collectively encountered and attacked per host-seeking mosquito per night [11, 24–30]
*A*_*h,p*_	Availability of all protected (*p*) human (*h*) blood hosts for attack, expressed as the rate at which they are collectively encountered and attacked while protected by an LLIN or other prevention measure per host-seeking mosquito per night [11, 24–30]
*C*_*A,p*_	Proportional coverage of all available blood resources that mosquito population utilizes (*A*) with a protective intervention (*p*) [11]
*Reformulation to predict impact based on coverage and utilization rates of resource subsets*	
*α*_*R*_	Utilization rate for an entire given resource (*R*, which may be specified as blood (*v*), resting sites (*r*), sugar (*s*) or any other resource mosquitoes use), defined as the rate at which individual mosquitoes attempt to utilize all forms of that resource per gonotrophic cycle
	Utilization rate for a defined subset of a given resource (*R*, which may be specified as blood (*v*), resting sites (*r*), sugar (*s*) or any other resource mosquitoes use) that can be identified and targeted with an intervention (*x*) in the field (*R*_*x*_), defined as the rate at which individual mosquitoes attempt to utilize the subset per gonotrophic cycle
	Utilization rate for a defined subset (*x*) of a given resource (*R*, which may be specified as blood (*v*), resting sites (*r*), sugar (*s*) or any other resource mosquitoes use) that can be identified and targeted with an intervention (*x*) in the field during the subset of times (*y*) when it can be effectively protected by a given intervention (*R*_*x,y*_), defined as the rate at which individual mosquitoes attempt to utilize that subset at times when it can be protected per gonotrophic cycle
	Utilization rate for a defined subset of a given resource (*R*, which may be specified as blood (*v*), resting sites (*r*), sugar (*s*) or any other resource mosquitoes use) that has been identified, targeted (*x*) and covered (*c*) with an intervention during the subset of times (*y*) when it can be effectively protected by a given intervention (*R*_*x,y,c*_), defined as the rate at which individual mosquitoes attempt to utilize that covered subset at times and places at which it can be protected per gonotrophic cycle
	Utilization rates for a defined subset of a given resource (*R*, which may be specified as blood (*v*), resting sites (*r*), sugar (*s*) or any other resource mosquitoes use) that has been identified, can be targeted with an intervention (*x*) and has been surveyed entomologically (*z*) in the field (*R*_*x,z*_), defined as the rate at which individual mosquitoes attempt to utilize that sample of that subset per gonotrophic cycle
*α*_*v*_	Utilization rate for all available blood resources (*v*), defined as the rate at which individual mosquitoes attempt to utilize any source of blood per gonotrophic cycle
	Utilization rate for a defined subset (*x*) of all blood resources (*v*) that has been identified and surveyed entomologically (*z*) in the field (*v*_*x,z*_), defined as the rate at which individual mosquitoes attempt to utilize that sample of that blood source subset per gonotrophic cycle
*b*	Mean lifetime total number of bloodmeals acquired per emerging mosquito [26]
*B*_*l*_	Mean mosquito biting rates experienced by individual livestock (*l*), defined as the number of bites per head per night
*B*_*h*_	Mean mosquito biting rates experienced by individual humans (*h*), defined as the number of bites per person per night [26, 30]
*C*_*R*_	Coverage of all available forms of a given resource (*R*) with a vector control intervention
	Coverage of all available forms of an identifiable, targetable subset (*x*) of a given resource (*R*) with a vector control intervention
	Coverage of the human subset (*x* = *h*) of all available blood sources (*v*) while they are indoors (*i*) with long-lasting insecticidal nets (*n*) [11, 26–30]
*E*	Emergence or recruitment rate of mosquitoes in a defined setting per night [24, 26–29, 31]
*h*	Humans
*i*	Indoors
*j*	Gonotrophic age, expressed as the number of gonotrophic cycles completed
*l*	Livestock
*λ*_*t*_	Relative availability of an individual mosquito traps (*t*) for attack by host-seeking mosquitoes attempting to utilize it as a source of blood, compared to a single unprotected human [27]
*M*	Absolute size of the mosquito population in a given setting, defined in terms of the number of individuals present
	Rate at which the mosquito population utilizes a defined, entomologically surveyed sample subset (*z*) of any identifiable and targetable subset (*x*) of a given resource (*R*_*x,z*_), expressed as the number of utilization attempt events per night
	Rate at which the mosquito population utilizes a defined, entomologically surveyed sample (*z*) of human (*h*) blood resources (*v*_*h,z*_), expressed as the number of utilization attempt events per night
	Rate at which the mosquito population utilizes a defined, entomologically surveyed sample (*z*) of livestock (*l*) blood resources (*v*_*l,z*_), expressed as the number of utilization attempt events per night.
	Mortality probability associated with exposure to an intervention-covered (c) form of a given resource (*R*) through a single utilization attempt event
*N*_*l*_	Number of livestock (*l*) living in a defined setting [24, 25, 27–29]
*N*_*h*_	Number of humans (*h*) living in a defined setting [24, 25, 27–29]
*N*_*h,z*_	Number of persons directly sampled by an entomological survey (*z*) of mosquitoes attacking human (*h*) hosts
*N*_*h,Ω*_	Number of persons residing in all houses sampled by an entomological survey (*Ω*) of mosquitoes attacking human (*h*) hosts
*N*_*t*_	Number of mosquito traps (*t*) present in a defined setting [27]
	Probability of a mosquito surviving all attempts to utilize intervention-covered forms of the targeted resource per gonotrophic cycle
*P*_*γ*_	Probability of a mosquito surviving all utilization attempt events for all resources per gonotrophic cycle [24, 26, 28]
*P*_*γ*,0_	Probability of a mosquito surviving all utilization attempt events for all resources per gonotrophic cycle in the absence of any intervention
*P*_*f*_	Probability of a mosquito surviving one full feeding cycle (*f*) [24, 26–29, 31]
	Proportion of human (*h*) blood (*v*) host exposure to mosquito bites that occurs indoors (*i*) in the absence of any protective intervention [11, 24, 26, 30, 32–38].
	Proportion of all available bloodmeals (*v*) that originate from a specific livestock (*l*) host species subset [25]
	Proportion of all available bloodmeals (*v*) that originate from the human (*h*) host species subset [11, 22–27, 30, 39–41]
*R*	The total availability of all forms of a given resource, which may be specified as blood (*v*), resting sites (*r*), sugar (*s*) or any other resource mosquitoes use, defined as the per night rate at which individual mosquitoes encounter and attempt to utilize that resource
*R*_*x*_	The total availability of a subset (*x*) of a given resource (*R* which may be specified as blood (*v*), resting sites (*r*), sugar (*s*) or any other resource mosquitoes use) that can be identified and targeted with an intervention, defined as the per night rate at which individual mosquitoes encounter and attempt to utilize that subset
*R*_*x,y*_	The total availability of a subset (*x*) of given resource (*R* which may be specified as blood (*v*), resting sites (*r*), sugar (*s*) or any other resource mosquitoes use) that can be identified and targeted with an intervention during the subset of times (*y*) when it can be effectively protected by that intervention, defined as the per night rate at which individual mosquitoes encounter and attempt to utilize that subset at times when it can be effectively covered with that intervention
*R*_*x,y,c*_	The total availability of all intervention-covered (*c*) forms of a targetable subset (*x*) of given resource (*R* which may be specified as blood (*v*), resting sites (*r*), sugar (*s*) or any other resource mosquitoes use) during the subset of times (*y*) when it can be effectively protected by that intervention, defined as the per night rate at which individual mosquitoes encounter and attempt to utilize the covered forms of that subset at times when it is effectively covered with that intervention.
*R*_*x,z*_	The total availability of an entomologically surveyed sample (*z*) of a targetable subset (*x*) of given resource (*R* which may be specified as blood (*v*), resting sites (*r*), sugar (*s*) or any other resource mosquitoes use), defined as the per night rate at which individual mosquitoes encounter and attempt to utilize it.
*r*	The total availability of all forms of resting sites, defined as the rate at which individual mosquitoes encounter and attempt to utilize resting sites per night
*s*	The total availability of all forms of sugar, defined as the rate at which individual mosquitoes encounter and attempt to utilize sugar per night
*t*	Mosquito traps
*τ*	Mean number of nights individual mosquitoes spend resting and gestating indoors following a bloodmeal inside a house
*v*	The total availability of all forms of blood, defined as the rate at which individual mosquitoes encounter and attempt to utilize blood per night
*x*	A subset of a given resource that may be identified and targeted with a vector control intervention
*y*	A subset of a given resource that may be effectively covered with a vector control intervention at times and places when mosquitoes encounter and attempt to utilize it
*z*	A sample of a given resource that has been surveyed entomologically
*Ω*	Humans in a sampled set of households

### Defining biological coverage based on the example of blood resources

Biological coverage (*C*_*A,p*_) of all the blood resources upon which mosquitoes rely, with long-lasting insecticidal nets (LLINs) or any other personal protection measure, has been previously defined as the proportion of all mosquito attacks upon all available hosts for which those hosts were covered with a protective (*p*) intervention at that time and place [11, 24]:
1

where the total attack availabilities of the all hosts (*A*), and covered hosts at times and places when they are actually protected (*A*_*p*_), are defined kinetically [25, 42] as the rates per night at which an individual host-seeking mosquito respectively encounters and attacks [26] either all hosts or all hosts that are protected at the time of the encounter and attack events.

However, to allow simplified notation for generalization of this approach to a greater diversity of distinct resources, here the symbol *A* is replaced by *R* to denote the total kinetic availability rate of a specific given resource, which may be specified as blood (*v*), resting sites (*r*), sugar (*s*) or any other resource (*R* ∈ *v*, *r*, *s* …). Also, the terms *attack* and *protected* which were previously used to define availability in kinetic terms for models of blood resource seeking and acquisition [26, 27] are not entirely appropriate for non-blood resources, so these are replaced with more generally applicable terms *attempt to utilize* and *covered*, respectively. Furthermore, now that biological coverage has been defined to explicitly consider only protection that is in place at the times and places when that resource is utilized, *de facto* coverage (*c*) and protection (*p*) are equivalent to each other (*c ≈ p*), so the former is used to simplify and harmonize the notation. Expressing Equation 1 in terms of this revised notation yields:
2

where *C*_*v*_ is the proportion of all mosquito attacks upon real (live vertebrate hosts) or perceived (artificial odor-baited traps, sometimes referred to as pseudo-hosts [27]) blood resources to which effective coverage with a vector control intervention applies at that time and place, *v* is the total rate at which individual mosquitoes encounter and attack all hosts and pseudo hosts, and *v*_*c*_ is the total rate at which individual mosquitoes encounter and attack all hosts and pseudo hosts at times and places when they are effectively covered with a vector control intervention.

In the case of interventions such as LLINs, which only protect humans while they use them indoors, biological coverage can be calculated as the product of the proportion of all bloodmeals (*v*) that originate from the human (*h*) host species subset , the proportion of human exposure to mosquito bites that would otherwise occur indoors (*i*) without an LLIN , and the proportional *demographic* coverage of humans, measured as the proportion of humans who reported using a net while asleep indoors the previous night  [11]:
3

where all three terms on the right hand side of Equation 3 are defined as sequentially nested fractions and sub-fractions of the total availability of all blood hosts (*v*) that are represented by humans (*h*), those humans while indoors (*i*), and those humans while indoors and protected by coverage with LLIN use at the time (*c*):
456

### Adapting the concept of biological coverage to rationalize vector control impact based on utilization rates of diverse resource targets

Expressing Equation 2 in more general terms that may be applied to any given resource (*R*), rather than blood specifically (*v*), yields the following formula:
7

LLINs that directly kill mosquitoes when they encounter and attack protected human blood sources are the best established [43] and easiest resource targets to conceptualize and model, but previous formulations predicted their impact upon vector survival by assuming mosquitoes feed once and only once per gonotrophic cycle [26]. However, resting sites, oviposition sites, and even blood resources themselves, may be utilized more than once per gonotrophic cycle [44], while sugar sources may be used less than once [21, 45]. To adapt the concept of biological coverage to more diverse resource targets which are used more than once per gonotrophic cycle, the term *resource utilization* is defined as the mean rate at which mosquitoes utilize any given resource (*R*) per gonotrophic cycle (*α*_*R*_). This definition of resource utilization rate can be expressed mathematically as the product of the duration of the gonotrophic cycle, expressed as nights per gonotrophic cycle (*g*), and the rate per night at which a mosquito population utilizes that resource (*m*_*R*_), divided by the size of the mosquito population (*M*):
8

where *g* is expressed as units of nights, *m*_*R*_ in units of utilization attempt events per night, and *M* as the number of individual adult mosquitoes present in the population. For any targetable, intervention-covered (*c*) proportion of that resource (*R*_*c*_), the corresponding rate at which mosquitoes encounter and attempt to utilize that covered fraction , by definition, varies in proportion to the fraction of the kinetic availability of that resource that it represents:
9

Hence the quotient of the availability or utilization rates for the total resource, divided into those for the intervention-covered fraction, are equivalent to biological coverage of that resource:
10

Most vector control strategies only target a specific subset (*x*) of the resource that they are delivered to, which is practically definable, identifiable, accessible, and treatable in the field. Similarly to resource coverage (Equation 10), the proportion of all available forms of a specific resource (*R*) that is accounted for by a given subset (*R*_*x*_) of that resource , can be defined and measured in terms of the rate at which mosquitoes encounter and attempt to utilize it [26, 27, 42] by generalizing Equation 4 for subsets of any possible resource, rather than specifying blood:
11

Similarly, the proportion of that subset (*x*) that is effectively protected at times when it is utilized by mosquitoes (*y*) can be expressed in terms of the proportion of resource utilization attempt events it accounts for in that resource subset:
12

Hence, the biological coverage of all forms of that resource (*C*_*R*_) can be expressed more explicitly than in Equation 10 as the product of the proportion of that resource represented by that subset , the proportion of utilization attempt events for that subset to which protection effectively and conditionally applies  and measured intervention coverage of that resource subset at times and places when it may be effectively protected :
13

Note that  is the utilization rate of the covered fraction of the targeted resource subset, equivalent to  in Equation 10, because all covered forms of the resource occur amongst the intervention-targeted subset of that resource (*x*) at the times and places at which they mosquitoes actually attempt to utilize them (*y*):
14

### Predicting intervention impact based on resource subset coverage and utilization rates

Interventions targeting adult mosquitoes may have quite complex modes of action, repelling mosquitoes away from humans [46] or contaminating them with agents that affect their longevity [47, 48], competence [47, 48] or fecundity [49, 50]. Biological agents may be transmitted horizontally or vertically through the population [47, 48, 50], while coverage amplification of chemicals may be achieved by mosquito-mediated transfer between resources [49]. Regardless of their complexity, these diverse strategies can all be enhanced by maximizing biological coverage of the resource targeted to ensure maximum contact with the mosquito population, and this is a critically important determinant of success in its own right. Previous formulations describing mosquito survival and mortality as a function of exposure to LLINs or IRS [26] are therefore adapted and simplified as follows to allow for a range of utilization rates ranging from zero to several times per gonotrophic cycle, rather than the previously assumed utilization rate of once per gonotrophic cycle for all blood resources (*α*_*v*_=1). All predictions of impact upon mosquito survival, and the entomologic inoculation rates they mediate, were implemented and parameterized exactly as previously described [51], except that Equation 14 of the original formulation [26] was adapted to incorporate the mortality risks of utilizing all covered and uncovered resources in a more generally applicable manner:
15

where *P*_*γ*_ is the probability of surviving all utilization attempt events for all resources per gonotrophic cycle, *P*_*γ*,*0*_ is the probability of surviving all utilization attempt events for all resources per gonotrophic cycle in the absence of any intervention, and  is the probability of surviving all attempts to utilize intervention-covered forms of the targeted resources per gonotrophic cycle. The probability of surviving all attempts to utilize intervention-covered forms of the targeted resource per gonotrophic cycle  can be calculated as an exponential decay function of the product of the mortality probability associated with exposure to a covered form of the resource through a single utilization attempt event , and the mean utilization rate for all covered forms of that resource :
16

By substituting rearranged forms of Equation 10 and then Equation 13 into Equation 16, a solution with two field measurable parameters for the targetable, quantifiable, surveyable, subset is derived:
17

It is therefore not necessary to know the proportion of that total resource which the targeted subset represents, or the coverage (*C*_*R*_) or utilization rate (*α*_*R*_) for all available forms of a resource. Impact can be predicted directly so long as the coverage of the targeted subset itself , and utilization rates for that subset under conditions that enable the intervention to protect it against safe utilization by the mosquito , can be measured.

This approach to predicting the survival probability assumes that utilization attempt events are randomly, and independently, distributed across all resource units and mosquitoes. Specifically, the number of times one mosquito utilizes a resource (or resource subset) in one gonotrophic cycle is assumed to be a non-negative integer valued random variable (0, 1, 2, 3…) since the mosquito may not necessarily use the resource or, alternatively, may access it multiple times. Hence, the utilization rate of these resources should be understood as an expected value depending on random events that may be expressed as a mean. This is clearly not the case in relation to obligate utilization of blood from one of *all* available blood resources (*R* = *v*). Each mosquito must utilize one of these available resources to complete the gonotrophic cycle, so complete coverage of all blood resources (*C*_*R*_ = 1) that are utilized at a mean rate of once per gonotrophic cycle (*α*_*R*_ = 1) with an insecticide which induces comprehensive fatality  would deterministically result in reduction of survival probability to zero , rather than merely reduced to the minor proportion of mosquitoes that are inaccurately assumed by Equation 16 to have completed a gonotrophic cycle without taking any bloodmeal. However, for a covered *subset* of a resource (Equation 17), rather than for all available forms of that resource (Equation 16), it is realistic to assume that the number of utilization attempt events per gonotrophic cycle is a random variable for individual mosquitoes and utilization rates per gonotrophic cycle are expected values (expressed as a mean), even for obligate blood resource utilization behaviours.

### Measuring utilization rates for subsets of undefined resources by comparison with those for quantifiable blood resources

Adult mosquitoes use many distinct resources during their lifetimes, including several that they need afresh every time they complete a gonotrophic cycle: blood, resting sites, and oviposition sites. Most of these resources are difficult to quantify directly, so the same is true of the rates at which mosquitoes utilize them, thereby making contact with them. However, measurements of feeding rates upon humans or livestock allow ready quantification of absolute mosquito population size or recruitment rate [31]. This is because the size of these mammalian host populations can be conveniently measured by direct census, and blood acquisition occurs at a measurable rate per host [52] with a measurable probability for a given blood host species [22, 23]. Also, blood acquisition usually occurs at a utilization rate of only once per gonotrophic cycle (*α*_*v*_ ≈ 1) where sugar availability is not limiting [45], except for the first gonotrophic cycle where two bloodmeals may be required [53, 54]. For example, the emergence or recruitment rate of mosquitoes (*E*) in a given setting can be calculated as function of the measured mean biting rate experienced by individual humans (*B*_*h*_), the number of humans living there (*N*_*h*_), the proportion of bloodmeals obtained from humans , and survival probability per feeding cycle (*P*_*f*_) [31]:
18

where the mean lifetime number of bloodmeals per emerging mosquito (*b*) is calculated as the sum of the probabilities of surviving to all plausible gonotrophic ages, expressed as the number of gonotrophic cycles completed (*j*) [26, 31]:
19

Similarly, for a very zoophagic (predominantly animal-feeding) vector with a strong preference for a known, accessible, manageable non-human host species such as cattle, goats, sheep, pigs or other livestock (*l*), it may be easier to accurately measure biting rates upon such livestock (*B*_*l*_) so the equivalent calculation can be made if the proportion of blood obtained from that host species , and the population size of that host species (*N*_*l*_) can be determined:
20

The key to applying Equations 18 and 20 to estimate absolute vector population sizes is the assumption that the fraction of all available sources of blood that each entomologically surveyed host represents  can be readily estimated by host census and bloodmeal identification from a sample of resting, blood-fed mosquitoes, so it is not necessary to directly detect all biting events on all hosts. Generalizing this principle, the emergence rate of mosquitoes (*E*) can be estimated based on the rate at which mosquitoes are trapped or observed utilizing  a surveyed sample subset (*z*) of any targetable subset (*x*) of a given resource (*R*_*x,z*_) if the proportion of all available forms of that resource which that surveyed subset represents (*R*_*x,z*_*/R*), and the rate at which individual mosquitoes utilize all available forms of that resource per gonotrophic cycle (*α*_*R*_), are both known. Note also that equation 18 and 20 both also implicitly include a term in the denominator for the utilization rate of all blood sources, that was negated by assumed a value approximating unity (*α*_*v*_ ≈ 1) but can be explicitly reintroduced for the purposes of generalization. Substituting  for *B*_*h*_ or *B*_*l*_, *R*_*x,z*_*/R* for  or , and *α*_*R*_ for *α*_*v*_ in Equations 18 and 20, respectively, yields the following general formula:
21

Blood resources can be readily identified as discrete units and their total numbers can be quantified by head-count census. However, units of sugar, resting site, oviposition site, and mating site resources are difficult to define unambiguously, except where these are introduced artificially (sugar baits, houses, boxes, pots, barrier screens, water containers, or swarming markers), and the total quantity of these resources available in the environment is even more difficult, if not impossible, to ascertain. Therefore, it is not obvious how *R*_*x,z*_*/R* can be estimated for these resources with existing field survey methods. However, this is not necessary to know, because intervention impact can be conveniently rationalized in terms of coverage and utilization rates for definable, targetable subsets of those resources (Equation 17), and it is possible to calculate their relative rates of utilization compared with those for blood from a known proportion of all available blood resources. Here the quantifiable total blood resource, and a surveyed sample (*z*) of an identifiable subset (*x*) of that blood resource, is specified (*R = v*) and distinguished from equivalent terms for other resources, such as resting sites (*R = r*) or sugar (*R = s*), with the specific terms *v* and *v*_*x,z*_. Also, the per gonotrophic rates at which mosquitoes attempt to utilize (*α*) all blood resources (*v*) or a distinct, identifiable subset of blood resources (*v*_*x*_), as well as the per night rate at which utilization attempt events occur (*m*) at a surveyed sample (*z*) of that blood resource subset (*v*_*x*_), are distinguished from those for other resources with the specific terms , and , respectively. Given that resource utilization rate per gonotrophic cycle is proportional to the total rate at which utilization attempt events occur in the overall population (Equations 8, 9, 10 and 11), the relative rate of utilization of such a resource subset compared to all blood resources can be expressed by dividing Equation 21, which is specified for a given non-blood resource subset and a surveyed sample thereof (*R*_*x*_/*R*_*x*,*z*_), by an equivalent formulation specified for all blood resources, and a surveyed sample of hosts from a subset for which bloodmeals recovered from the midguts of recently fed specimens can be identified and distinguished from other sources (*v*/*v*_*x*,*z*_), and rearranging:
22

Note that the emergence and mean longevity terms cancel each other out so that estimates of these parameters are not required to estimate the relative rate of utilization of a resource compared with blood, as described below.

The most obvious vertebrate blood resource subsets (*v*_*x*_) which are readily surveyed, including detection of blood feeding events and identification of blood source in specimens of fed mosquitoes, are humans (*x = h*) and livestock (*x = l*) [52]. The proportion of all available blood resources sampled by the field survey can be quantified as the product of the proportion of bloodmeals obtained from humans  or livestock  and the number of humans (*N*_*h,z*_) or cattle (*N*_*l,z*_) sampled by the host attack survey, divided by the total number of humans (*N*_*h*_) or livestock (*N*_*l*_) present:
23

Fortunately, while gonotrophic discordance beyond the first feeding cycle does occur in *Anopheles*, it is unusual and can be quantified [45]. In most cases, it is therefore reasonable to explicitly assume that mosquitoes predominantly utilize blood approximately once per feeding cycle (*α*_*v*_ ≈ 1), so substituting the host-specified (*v*_*x*_ *= v*_*h*_ or *v*_*l*_) formula of Equation 23 into Equation 22, and replacing *α*_*v*_ with unity, yields a solution for  for which all the terms are measurable in the field:
24a

or
24b

where *R*_*x,z*_/*R*_*x*_ is the proportion of all available forms of the targeted non-blood resource subset that was surveyed entomologically to measure the rate per night at which the entire mosquito population attempts to utilize it , where *N*_*h,z*_/*N*_*h*_ and *N*_*l,z*_/*N*_*l*_ are the proportions of all humans or livestock that were respectively surveyed to measure the rate at which mosquitoes attempted to utilize their blood, and where  and  are the proportions of bloodmeals the vector population obtains from all available humans and livestock, respectively.

Where two resources co-occur and overlap completely with each other, specifically the example of resting sites (*R* = *r*_*i*_) and human blood indoors within houses (*v*_*h,i*_), the proportion of each resource subset that is sampled is no longer required because these cancel each other out. All that is required is an estimate of the number of persons or person nights sampled by the host attack survey (*N*_*h,z*_), and the total number people staying in those sampled houses (*N*_*h,Ω*_), or even their ratio, which is commonly referred to as the mean number of occupants per house (*N*_*h*,*Ω*_/*N*_*h*,*z*_):
25a

In some experiments, however, resting events following more than one bloodmeal are represented in surveys of resting sites because those events may last two or more days. Recent standardized trials to compare various techniques for catching host-seeking and resting mosquitoes [55–57] placed these in or immediately outside of different houses within a defined sampling frame each night, so that the former would not compete with the latter by trapping mosquitoes before they can feed and rest. Also, spray catches must be spaced by intervals of several days to allow residual pyrethrum to dissipate. In both cases, mosquitoes gestating over two or more preceding nights are allowed to accumulate from multiple nights of blood feeding (*τ*) in the surveyed houses, and this must be accounted for when estimating utilization rates:
25b

### Literature review and utilization rate estimate extraction

Studies, or sets of studies, were identified which presented sufficient parameter estimate data for utilization rates of specific, intervention-targetable resource subsets to be calculated for specific malaria vector species in specific, distinct locations. In addition to the authors’ archives of literature and unpublished data, the Pubmed database was also queried with the search term ‘*Anopheles* AND ((pyrethrum spray OR aspirator) OR (insecticide AND (cattle OR livestock)) OR odour-baited OR sugar)’. For utilization of livestock blood and sugar, consideration was limited to studies in settings where trials of insecticide-treated livestock or sugar baits, respectively, have been either implemented or specifically suggested. To avoid cluttering of the second figure presented in the Results section, consideration of studies enabling estimation of indoor resting site utilization was restricted to recent unpublished studies of our own and those published in the last decade. Where results for a species complex or group were reported, these are attributed to the most common sibling species identified in that population.

Utilization rates for blood from humans while indoors, when they can be protected with LLINs, was calculated as the product of the proportion of human exposure to mosquito bites occurring indoors  and the human blood index , by assuming a single bloodmeal per gonotrophic cycle:
26

Where local estimates for the proportion of bloodmeals obtained from humans  were not available for the vector in question, the median values for that species from a previous review [39] were applied. Where direct estimates of the proportion of bloodmeals obtained from cattle and other treated livestock  were not available, utilization of blood from other non-human sources was assumed to be negligible, so that this quantity could be calculated as the complement of the proportion obtained from humans :
27

Utilization rates for odour-baited traps are calculated by assuming that the probability of a mosquito attacking a trap, rather than a natural host, per gonotrophic cycle is equivalent to the proportion of all available human hosts, animal hosts, and pseudo-hosts, that they represent [27]. This is calculated on the basis of the ratio of traps to people (*N*_*t*_*/N*_*h*_), the relative availability of those traps (*λ*_*t*_), and the proportion of bloodmeals obtained from non-human hosts  [27]:
28

Utilization rates for sugar resource subsets  were calculated as follows, based on direct field measurements of utilization rates per day for dye-labelled sugar baits  [18–20], and an assumed mean gonotrophic cycle duration of three days (*g = 3*):
29

Utilization rates for resting site subsets , such as the insides of houses or artificial shelters and netting barriers placed in or around them, were estimated using Equation 25a or 25b based on the quotient of the mean rate at which mosquitoes were caught resting inside a sample of them (*m*_*r,x,z*_) adjusted, where necessary, for an assumed indoor resting period of 2 days (*τ* = 2), divided by the rate at which they were caught attacking human hosts indoors , the mean number of exposed occupants per house or room (*N*_*h,Ω*_/*N*_*h,z*_) adjusted for reported usage rates of LLINs  which were assumed to confer complete protection, the proportion of bloodmeals obtained from humans , and the proportion of human bloodmeals obtained indoors , with the latter two parameters usually assumed from mean literature values reported for that species [32, 39]:
30

## Results

### Dependence of impact upon utilization rates of intervention-targeted resource subsets

**Figure 1:**
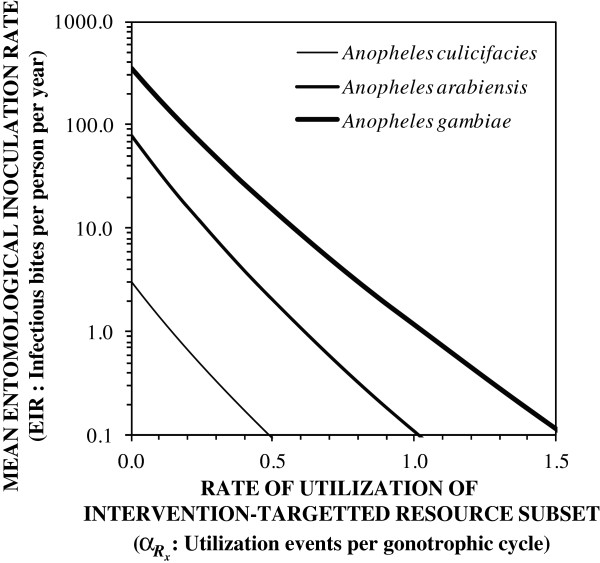
**The predicted relationship between the impact of vector control upon malaria transmission and the rate at which mosquitoes utilize the resource subset targeted by the intervention**  **.** *Anopheles epiroticus*, *An. arabiensis*, and *An. gambiae* were chosen as examples of vector species that feed primarily upon animals, on both humans and animals, or primarily upon humans. Zero coverage of the human blood resource with LLINs  and high biological coverage of the resource subset which is targeted with an intervention  that induces high mortality among mosquitoes that utilize covered forms of that resource  were assumed. Apart from the adaptation of the original formulation to capture intervention impact upon vector survival as a generally applicable function of target resource subset coverage and utilization rate (Equation 15 to 17), all predictions were made by parameterizing and executing deterministic models of malaria transmission and control, exactly as previously described [26, 51, 62].

Figure 1 illustrates how the impact of a vector control intervention with a mosquito-toxic active ingredient depends on the utilization rate of the resource subset to which it is targeted, and on the pre-existing level of transmission mediated by the vector. Utilization rates exceeding, or at least approaching, one event per gonotrophic cycle are required to achieve useful reductions of intense, saturating transmission [4, 58] mediated by human-feeding mosquitoes such as *Anopheles arabiensis* and *Anopheles gambiae*. Less efficient vectors that predominantly feed upon animals, such as *Anopheles culicifacies,* cause far lower levels of transmission and morbidity burden [39] that can respond more sensitively to effective transmission control because baseline exposure levels are not sufficient to saturate the human population with parasite infections [24, 58–61]. Very valuable impact upon transmission by such zoophagic mosquitoes may even be achieved by targeting resource subsets that are used as rarely as once every three feeding cycles because most malaria is transmitted by mosquitoes that are at least four gonotrophic cycles old [31, 54]. However, unless a resource subset is used at least once in every five gonotrophic cycles, it is unlikely to be a useful target for suppressing mosquito survival and population density through vector control, even for weak zoophagic vectors that rarely feed upon humans and mediate modest, manageable levels of transmission.

### Field estimates of utilization rates for defined, targetable resource subsets

**Figure 2:**
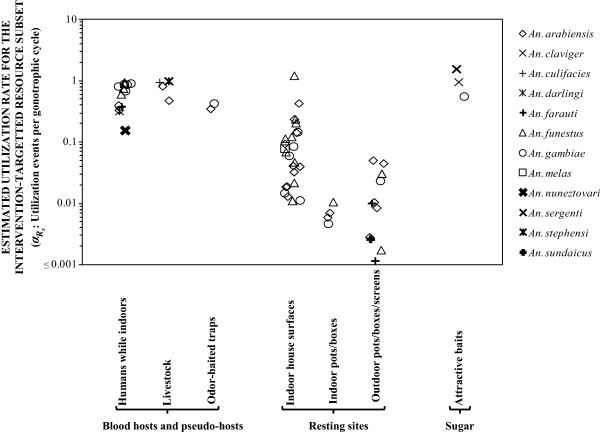
**Estimated utilization rates for a variety of blood, resting site and sugar resource subsets**  **based on existing field data.** See Additional file 1 for details of literature, parameter values and equations used to calculate the estimates presented.

Figure 2 illustrates the range of utilization rate estimates that could be extracted from the literature for a diversity of resource subsets used by mosquitoes. The predictions of Figure 1, and the generally high utilization rates of human blood while people are indoors (Figure 2), are approximately consistent with the documented impacts of LLINs upon malaria transmission [43]. It is also notable that the lowest estimates for utilization of human blood indoors (Figure 2) are for *An. arabiensis*, *Anopheles farauti*, *Anopheles darlingi*, and *Anopheles nuneztovari*, all of which have been known to persist and dominate residual transmission systems following scale-up of LLINs or IRS [6–8, 33]. Insecticide-treated livestock also appear to be as promising a target for zoophagic vectors as LLINs are for anthropophagic (predominantly human-feeding) vectors, and the utilization rates estimated for *Anopheles culifacies* and *Anopheles stephensi* are consistent (Figure 2) with the proven impact of this approach upon malaria transmission by these species [63]. Odour-baited traps act as pseudo-hosts by mimicking, and even surpassing [64], the taxis stimuli of normal blood sources for mosquitoes, luring them to fatal trap devices or insecticide-treated surfaces [27]. While these can achieve useful utilization rates if their attractiveness and positioning can be optimized, these estimates are somewhat lower than for insecticidal nets and livestock treatments directed at the best-matched vector species (Figure 2) because these devices must compete with natural hosts that, therefore, constitute an inevitable gap in biological coverage [27].

Apart from blood, the other important nutrition source that facilitates mosquito survival and malaria transmission is plant-derived sugar [21, 65, 66]. Estimated minimum utilization rates for dye-labelled sugar baits by *Anopheles claviger* [19], *Anopheles sergenti* [18], and *An. gambiae* [20], in three distinct settings appear to be at least comparable with those for utilization of human blood indoors by very anthropophagic vectors, and for utilization of animal blood by zoophagic vectors (Figure 2). The impressive impacts upon all three of these vector populations that have been achieved by adding insecticides to such sugar baits [18–20] are therefore consistent with the predictions outlined in Figure 1, as well as other recent modelling analyses [67]. Given the widespread dependence of mosquitoes upon sugar [21, 65, 66], many important vector populations probably use it at similarly high rates, especially when infected with malaria parasites [68]. Mosquitoes should therefore be at least as amenable to control with this approach as anthropophagic vectors are to LLINs [43], and as zoophagic vectors are to insecticide-treated livestock [63].

Utilization rate estimates for indoor resting sites (Figure 2) are generally lower than that required to explain (Figure 1) the often massive impact of IRS [13] and insecticidal wall linings [69] on many target vector species. However, this is not entirely surprising because even the best techniques for capturing mosquitoes resting indoors, such as pyrethrum spray catch [52] and backpack aspirators [70], are known to consistently under-sample them. Nevertheless, the estimates of resting site utilization presented in Figure 2 are clearly useful for comparison of different potential resting site targets, confirming that comprehensive spraying or lining of entire rooms and houses is probably superior to targeted treatment of pots, boxes (some of which were baited with host odours) or screening barriers, all of which were placed indoors for endophilic vectors or outdoors for exophilic ones [13–17]. However, perhaps the most important observation in relation to these estimates of resting site subset utilization rates is that they rely on upon quite complicated calculations requiring at least five distinct input parameters (Equations 26a, 27b and 30), many of which have to be assumed based on best guesses or literature values from a different setting (Additional file 1). In fact, none of the estimates presented in Figure 2 are based entirely upon local estimates for all the input parameters (Additional file 1), and are therefore not entirely independent of each other or representative of the full range of values for any of the vector species described.

## Discussion

### Defining and surveying targetable resource subsets

Estimating coverage and utilization of a resource subset primarily depends upon defining it in a quantifiable manner that can be readily surveyed and targeted, or artificially created in the field. The most obvious and familiar examples are the human populations targeted for universal coverage with LLINs to protect the blood resource they represent to mosquitoes [28, 43, 71]. While wild animals are difficult to survey or deliver interventions to, livestock represent blood resources that can be readily quantified and targeted with interventions [63]. It is even easier to track numbers and functionality of artificial odour-baited traps, which mimic and compete with natural blood sources for the attentions of host-seeking mosquitoes, so their potential impact can also be predicted as a function of biological coverage of all available hosts and pseudo-hosts [27].

The subset of all resting sites represented by the inner surfaces of human dwellings (walls, ceilings and even furniture) are the defined target for IRS [12, 71], as well as insecticide-treated tents [72], shelters [73, 74], and wall linings [69], so coverage can be quantified as the proportion of residential structures treated. On the other hand, artificially introduced pots, boxes, curtains, linings or screening barriers compete with natural resting sites [13–17]. Mosquitoes can be captured relatively efficiently on these well-defined, convenient, standardized surfaces, so it has been suggested that these could also be treated with toxic insecticides to improve control efficiency [13–17]. While it remains difficult to consistently identify and define sugar, oviposition site or mating site resources [5], recent progress with observational [75, 76], trapping [77], tracing and labelling [18–20, 22, 23, 78–81] methods for mosquitoes is encouraging.

### Adapting entomological survey techniques to measure resource utilization rates

Comparing the range of utilization rates described in Figure 2 with the predictions of potential impact illustrated in Figure 1 confirms that, despite their known limitations [52, 82], existing entomological field methods may be very useful for designing and evaluating a wide diversity of vector control products [83]. Both blood and sugar meals can be readily identified using a variety of naturally-occurring markers and artificially added tracers [18–20, 22, 23, 78–81], thus enabling very direct, robust measurement of label uptake as a function of time or age. Utilization rates can therefore be estimated directly for subsets of these naturally occurring resources (Equations 26, 27, and 29) or indirectly for artificially introduced subsets such as odour-baited traps (Equation 28).

Utilization rates for resting site subsets (Equation 30), or indeed any other non-blood resource (Equation 25a and 25b), can also be estimated indirectly by calibrating against measurable utilization rates for quantifiable, preferred blood sources. However, the complexity of these models, and their reliance upon local measurements of several entomological input parameters, all of which have limited precision and accuracy, may well limit broader application of this approach beyond the crude application to existing data presented in Figure 2. Recent attempts to rejuvenate and improve existing entomological survey methodology for detecting resource utilization attempt events with electrified grids [84–86], sticky traps [77, 87], mechanized aspirators [70], and high resolution cameras [75, 76], should enable improved sensitivity of utilization attempt event detection at surveyed samples of resource subset targets. However, while such technical advances may well address the inaccuracies of attempts to estimate utilization rates for subsets of resting sites or other non-nutritional resources by improving event detection sensitivity, they are unlikely to improve their precision because considerable uncertainty arises from the need for relatively complex models (Equations 25a, 25b and 30) that require correspondingly numerous measurements of input parameters.

Fortunately, a wide range of more sensitive chemical, biochemical, genetic and biological markers, that could be applied to labelling mosquitoes when they use these other resources, are now available [82] but these remain to be fully exploited. In fact, field studies using artificial tracers to label of both mosquitoes feeding upon sugar [18–20] and sand flies feeding upon rodent blood [88, 89], in which addition of insecticide removed almost all labelled insects from these vector populations, clearly demonstrate the validity of this strategy as a means to estimate biological coverage or utilization rates. The major advantage of labelling mosquitoes when they utilize a resource subset, rather than trapping or observing them, is that the measured proportions of marked insects can be readily analyzed with robust off-the-shelf statistical methods for binary outcomes, and are relatively precise because they have a nominator and denominator which both vary in proportion to population size or event detection sensitivity. The largest caveat to this approach is that essentially all targeted forms of that resource must be labelled on geographic scales large enough to negate the effect that mosquito dispersal has upon measurements of label uptake: immigration of unlabelled mosquitoes into the study area will increase the denominator while emigration will reduce the nominator, so that true local utilization rates will be systematically underestimated [52, 90, 91]. However, this phenomenon could also be exploited to great advantage if multiple distinct labels for various treatment arms were used to measure, and correct for, the effects of mosquito upon impact distribution in large-scale trials of vector control interventions [92].

The conceptual framework and entomological measurement priorities outlined here should be readily and directly applicable to almost any population of mosquitoes, vectors or other pest. It should therefore be possible to simultaneously tackle multiple vectors with integrated vector management programmes [93] that prioritize interventions based on simultaneous, comparative field assessment of respective utilization rates for each potential target species. Recent demonstrations of the usefullness of dummy bait products containing appropriate labels but no insecticide [18–20, 88, 89] illustrate how cost-effective, robust measurements of utilization rates could be used to select and optimize available technologies for immediate use or new prototypes for development.

## Conclusions

The concept of biological coverage can be extended to enable prediction of intervention impact for diverse vector control strategies based on estimated utilization rates for any definable, targetable resource subset. Indeed the applicability of this approach has been demonstrated here using existing entomological measurement methods to rationalize the observed impacts of LLINs, insecticide-treated livestock, and attractive toxic sugar baits upon malaria vectors. The development of improved and diversified technologies for controlling transmission of malaria, as well as a diversity of other vector-borne pathogens, could therefore be accelerated, rationalized and streamlined based on field measurements of the rates at which mosquitoes utilize targetable biological resource subsets.

While blood and sugar consumption can be readily quantified using existing methods for detecting natural markers or artificial tracers, improved techniques for labelling mosquitoes will be required to assess and optimize vector control measures which target them when they utilize resting, oviposition and mating sites. All mosquito species need sugar, resting sites, oviposition sites, and mating sites, as indeed do most arthropods of medical and veterinary importance. These resources are therefore important potential targets for the new or improved vector control methods that are clearly needed to eliminate malaria, and also a variety of other vector-borne pathogens. To enable comparative assessment of all potential resource subset targets, including sites which mosquitoes rest, oviposit or mate at, existing tracer technologies need be adapted to enable reliable, non-toxic, non-disruptive labelling of mosquitoes when they utilize these non-nutritional resource subset targets.

## Electronic supplementary material

Additional file 1: **Data sources, publications and calculations supporting Figure** 2 **.** (XLS 54 KB)
